# Iberian pig adaptation to acorn consumption: I. Net portal appearance of metabolites

**DOI:** 10.7717/peerj.5861

**Published:** 2018-10-31

**Authors:** Ignacio Fernández-Fígares, Jose Miguel Rodríguez-López, Lucrecia González-Valero, Manuel Lachica

**Affiliations:** 1 Department of Physiology and Biochemistry of Animal Nutrition, Estación Experimental del Zaidín, Consejo Superior de Investigaciones Científicas, Granada, Spain; 2 Départment Sciences Agronomiques et Animales, Institut Polytechnique LaSalle Beauvais, Beauvais, France

**Keywords:** Portal-drained viscera, Net portal appearance, Metabolites, Acorn, Pigs

## Abstract

Most valuable cured products from Iberian pigs come from pure bred animals raised for a final grazing-fattening period where pigs eat mainly acorns, a low protein energy rich fruit. This is a nutritional challenge for animals fed equilibrated diets from weaning. The aim of the study was to determine net portal appearance (NPA) of metabolites in gilts fed acorns and evaluate adaptational changes after one week of feeding. Two sampling periods were carried out (after one day and after one week of acorn feeding) with six gilts (34 kg average BW) set up with three catheters: in carotid artery and portal vein for blood sampling, and ileal vein for para-aminohippuric acid (PAH) infusion to measure portal plasma flow (PPF). Pigs were fed at 2.5 × ME for maintenance a standard diet in two portions, at 09:00 (0.25) and 15:00 h (the remaining 0.75). On the day prior to the first sampling period, pigs were fed 2.4 kg of oak acorns. After feeding 0.25 of ration a 6 h serial blood collection was initiated. Following an identical protocol, a second sampling session was performed 1 week later. Adaptation to acorn consumption decreased NPA of ammonia (47%, *P* < 0.001). Although there was a transfer of urea from the gastrointestinal tract to the circulation in both sampling periods, no differences in NPA of urea was found (*P* > 0.05). NPA of glucose was not influenced by sampling period (*P* > 0.05), but NPA of lactate was greatly increased (231%, *P* < 0.001). There was a negative NPA of albumin although adaptation to acorn feeding did not alter it. Although NPA of triglycerides and cholesterol were unchanged, a subtle increase in arterial and portal cholesterol was noticed (9.6%, *P* < 0.01). Pigs fed a protein deficient diet for one week adapted decreasing NPA of ammonia for saving metabolic energy as less ammonia would become available for conversion to urea.

## Introduction

The Iberian pig is an autochthonous breed from Spain and Portugal, and represents the most important Mediterranean swine type due to its population size and economic value. Traditionally, Iberian pigs have been raised extensively on seasonally available food resources producing meat products of the highest quality (López-Bote, 1998). Nowadays, to improve productivity, a semi-extensive system with balanced mixed diets is followed by a finishing phase under free range conditions up to 160 kg body weight (BW) in fall and winter. The Iberian pigs are about 100 kg BW at the beginning of the finishing phase in the oak woodland area and are allowed to graze consuming mainly fallen acorns from oak trees (approximately 0.84 of metabolizable energy (ME; García-Valverde et al., 2007), or 0.90 of dry matter (Rodríguez-Estévez et al., 2009) intakes) complemented with pasture when available. Although acorns are an energy rich resource, previous research in our laboratory has shown a shortage in protein supply and an imbalance in the provision of amino acids—mainly lysine—when acorns are the single feed available for the pig (Nieto et al., 2002; García-Valverde et al., 2007).

Portal-drained viscera (PDV) have a disproportionate influence on whole body metabolism which may affect the productivity of peripheral tissues by reducing available nutrients for other tissue components. Furthermore, splanchnic tissues contribution to total BW was greater in Iberian than Landrace pigs (Rivera-Ferré, Aguilera & Nieto, 2005) which emphasizes the importance of PDV for nutrient availability in Iberian pigs. The shift from a balanced diet to an acorn diet is an abrupt nutritional change with unknown consequences for the metabolism of Iberian pigs in the finishing phase.

We hypothesized that adaptational changes would occur at the PDV level after exclusive feeding of acorns to Iberian pigs. The aim of our study was to determine net portal appearance (NPA) of metabolites in Iberian gilts fed acorns from holm oak (*Quercus rotundifolia)* and evaluate if an adaptation to acorn feeding occurred after one week of consumption. In this manuscript, NPA of metabolites is presented and discussed while a second manuscript submitted (Fernandez-Figares et al., in preparation) will address NPA of amino acids.

## Methods

### Animals, facilities, and diets analysis and composition

The study protocol was approved by the Bioethical Committee of the Spanish Council for Scientific Research (CSIC, Spain; project reference RECUPERA 2020, FEDER funding).

Six Iberian (Silvela strain; Sánchez Romero Carvajal, Jabugo S.A., Puerto de Santa María, Cádiz, Spain) gilts of similar BW (25 ± 0.4 kg initial BW) were utilized. One week before surgery, gilts were housed in individual pens in a controlled environment room (21 ± 1.5 °C), with *ad libitum* access to water and a standard barley-soybean meal diet (145 g crude protein (CP)/kg dry matter (DM) and 14.3 MJ ME/kg DM). After surgery, pigs were placed in metabolic cages and fed the standard diet 2.5 × ME for maintenance (422 kJ/kg^0.75^ BW/d; Nieto et al., 2012) in two portions, at 09:00 (0.25) and 15:00 h (0.75). The day before the first sampling, the standard diet was substituted for a non-supplemented acorn diet and continued until the end of the experiment. The acorn diet consisted of 2.4 kg of acorns providing 1.425 kg DM and a total CP intake of 74.1 g. The Iberian pig shows the particular feeding behaviour whereby only the inner kernel is consumed and the hull is discarded. Composition and chemical analysis of the pre-experimental diet was performed by standard procedures (AOAC, 2000) and can be found elsewhere (González-Valero et al., 2016b). The nutrient composition of the acorn kernels fed is shown in Table 1. DM (no. 934.01), ether extract (no. 920.39) and total ash (no. 942.05) analysis were performed by standard procedures (AOAC, 2000). Total N was determined according to the Dumas’ method, by total combustion in TruSpec CN equipment (Leco Corporation, St. Joseph, MI, USA) and CP is defined as total N × 6.25. The neutral, acid and lignin detergent fractions (aNDFom (NDF assayed with a heat stable amylase and expressed exclusive of residual ash), ADFom (ADF expressed exclusive of residual ash) and Lignin(sa) (lignin determined by solubilization of cellulose with sulfuric acid), respectively) in kernels were analyzed by the method of Goering & Van Soest (1970). Neutral and acid detergent fibre was determined using an ANKOM^220^ Fibre Analyser Unit (ANKOM Technology Corporation, Macedon, NY, USA). Gross energy was measured in an isoperibolic bomb calorimeter (Parr Instrument Co., Moline, IL, USA).

**Table 1:** Nutritional composition of the kernel of acorns used in the study (g/kg DM).

Ash	18
Organic matter	982
Fat (ether extract)	65.0
Nitrogen	8.3
CP	52.0
aNDFom	41.8
ADFom	16.7
Lignin(sa)	2.6
Total amino acids	45.5
Gross energy (MJ/kg DM)	15.1

### Experimental procedures, schedules and calculations

The day before surgery (28 kg average BW) pigs were fasted and water was removed. Catheters were surgically placed in carotid artery and portal vein for blood sampling, and in ileal vein for infusion of para-aminohippuric acid (PAH; 2% w/v; Sigma-Aldrich Química S.A., Madrid, Spain) which is used as a marker to measure blood flow. Detailed description of design, construction and maintenance of the catheters, surgical procedures and post-surgical care of pigs was previously described (Rodríguez-López et al., 2013).

Pigs were adapted to close contact with the staff involved with sampling procedures to minimize stress. Two sampling periods were established and carried out under identical conditions after pigs completely recovered from surgery. The standard diet was changed to a non-supplemented acorn diet (34 kg average BW) and the first sampling (sampling period 1) was initiated one day after the diet change; the second sampling (sampling period 2) began 7 days later.

On the day of sampling, an initial 300 mg pulse dose of PAH was administered into the ileal vein 45 min prior to blood collection, and followed by a continuous infusion of 16 mg/min according to Yen & Killefer (1987), using a syringe pump (Model 33, Harvard Apparatus Inc., Holliston, MA, USA). Apyrogenic filters (MILLEX GP, Syringe Driven Filters Unit, 0.22 µm; Millipore, Carringtwohill, Ireland) were fitted to infusion syringes. Blood samples collected in 4.5 mL (Monovette VetMed; Sarstedt, Nümbrecht, Germany) heparinized tubes were taken simultaneously from carotid artery and portal vein −5 min, 0.5, 1, 1.5, 2, 2.5, 3, 3.5, 4, 5 and 6 h after feeding 0.25 of total daily acorn ration. Haematocrit was determined using a microcentrifuge (11,500 × g for 5 min; Biocen, Orto-Alresa, Ajalvir, Madrid, Spain). Plasma was obtained by centrifugation (4 °C, 1,820 × g for 30 min; Eppendorf 5810 R, Hamburg, Germany) and stored in aliquots at −20 °C for analysis of metabolites (ammonia, urea, glucose, lactate, albumin, creatinine, triglycerides and cholesterol) and PAH. After sampling, pigs were fed the remainder of the daily acorn ration and received 2.4 kg of acorn diet per day for one week after which the second sampling period (period 2) was carried out following identical protocol.

Portal blood flow (PBF) and portal plasma flow (PPF) were determined by the indicator dilution method using portal vein haematocrit and plasma PAH concentrations (Katz & Bergman, 1969). The PBF and NPA of metabolites were calculated according to the Fick principle of arterio-venous concentration difference and flow rate (Zierler, 1961). The PPF was calculated as infusion rate of PAH/(portal plasma PAH concentration − arterial plasma PAH concentration); PBF was calculated as PPF/[1 − (haematocrit/100)]. The NPA of a nutrient or metabolite was calculated as PPF × (portal concentration − arterial concentration). Positive NPA indicates production or release, whereas negative values indicate uptake or transfer of the substance.

### Statistical analyses

The experimental unit was the pig. Measurements were made sequentially over time in the same experimental unit. Postprandial data were subjected to multivariate ANOVA analysis using the MIXED procedure of the Statistical Analysis Systems Institute (SAS, 2002) for repeated measures. The main effects in the model were sampling period (1 vs. 2), sampling time (from 0–6 h) and their interaction. Differences were considered significant when *P* < 0.05.

## Results

No difference in the time needed to eat 0.25 of daily ration was found between sampling periods. Based on our observations in 105 kg BW Iberian pigs (García-Valverde et al., 2007) and the composition of the acorns used in this study, we assume that 600 g of acorns provided about 254 g of digestible carbohydrates and 19 g of digestible lipids; it also supplied 5.4 g of true absorbed protein. It was estimated that 0.83 of the organic matter digested from the acorn was absorbed by the ileum.

**Figure 1:**
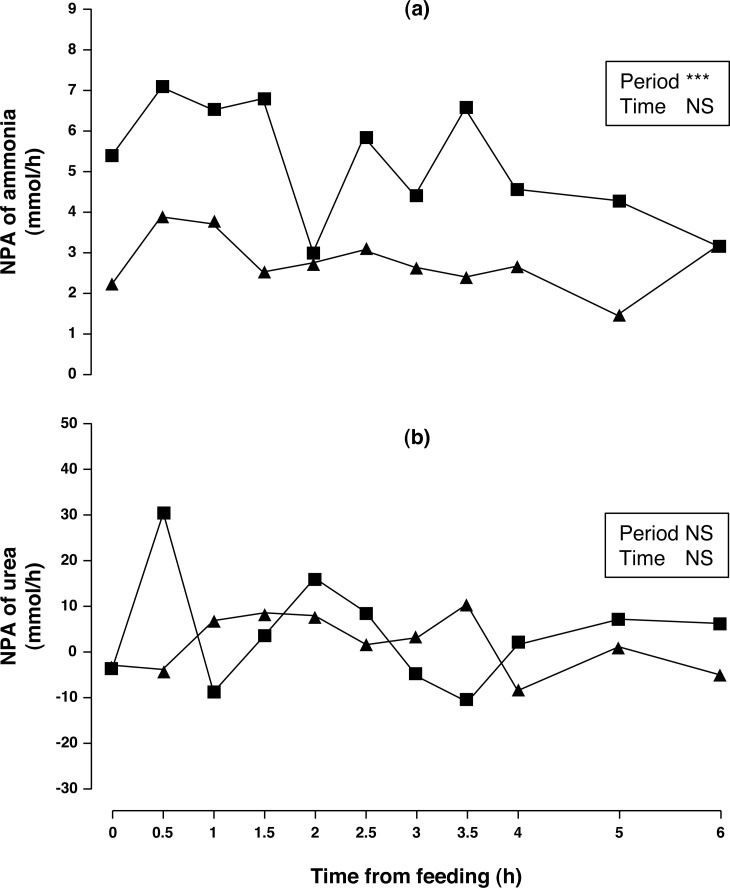
Net portal appearance (NPA) of ammonia (A) and urea (B) along a 6 h sampling in Iberian pigs (*n* = 6) fed acorns for 1 and 8 days (period 1 (■) and 2 (▴), respectively). *** *P* < 0.001; NS, not significant (*P* > 0.10).

**Figure 2:**
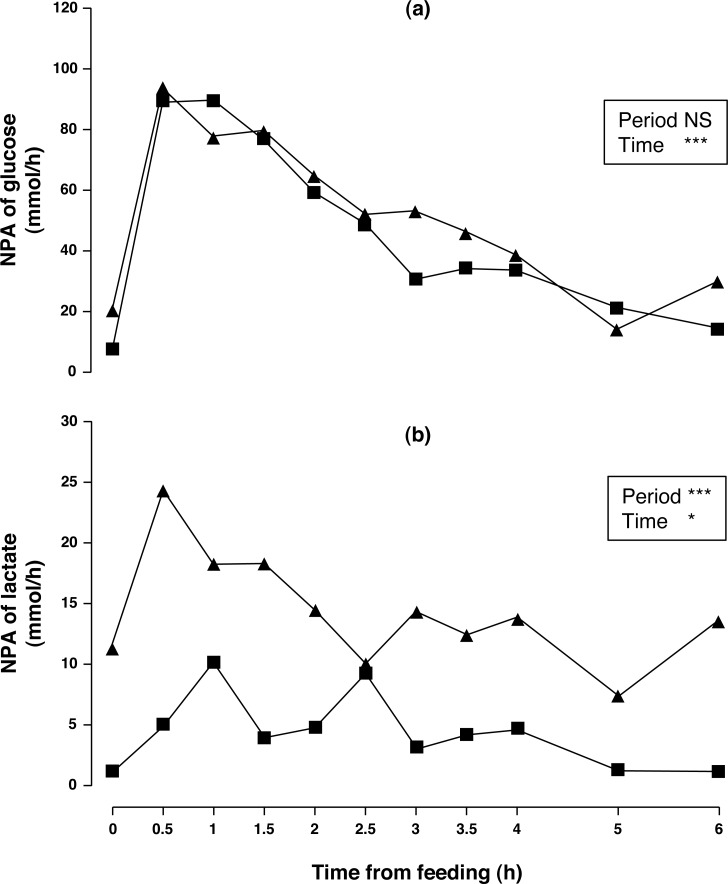
Net portal appearance (NPA) of glucose (A) and lactate (B) along a 6 h sampling in Iberian pigs (*n* = 6) fed acorns for 1 and 8 days (period 1 (■) and 2 (▴), respectively). * *P* < 0.05; *** *P* < 0.001; NS, not significant (*P* > 0.10).

**Figure 3:**
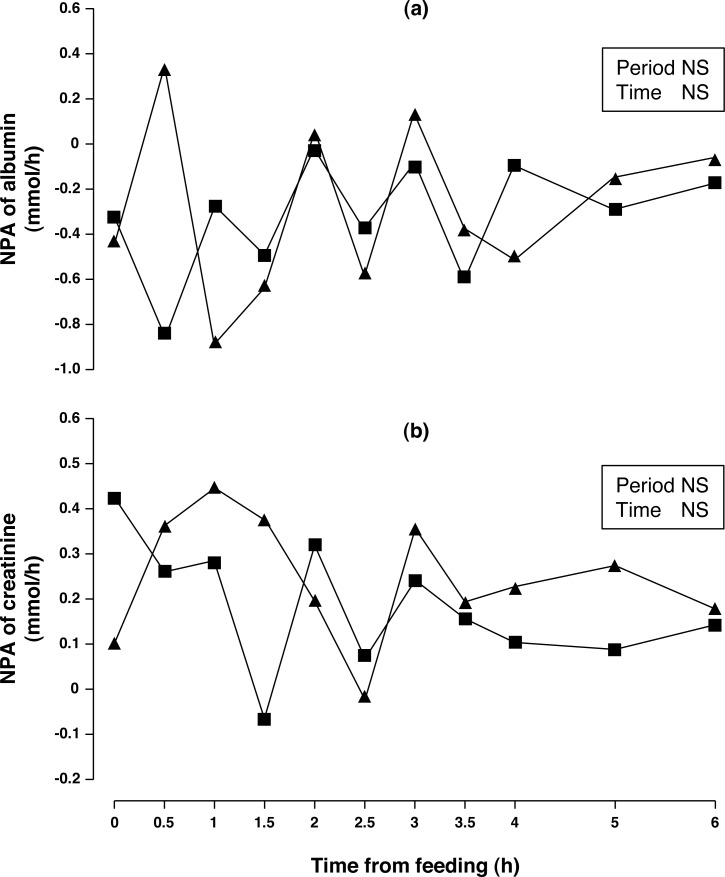
Net portal appearance (NPA) of albumin (A) and creatinine (B) along a 6 h sampling in Iberian pigs (*n* = 6) fed acorns for 1 and 8 days (period 1 (■) and 2 (▴), respectively). NS, not significant (*P* > 0.10).

**Figure 4:**
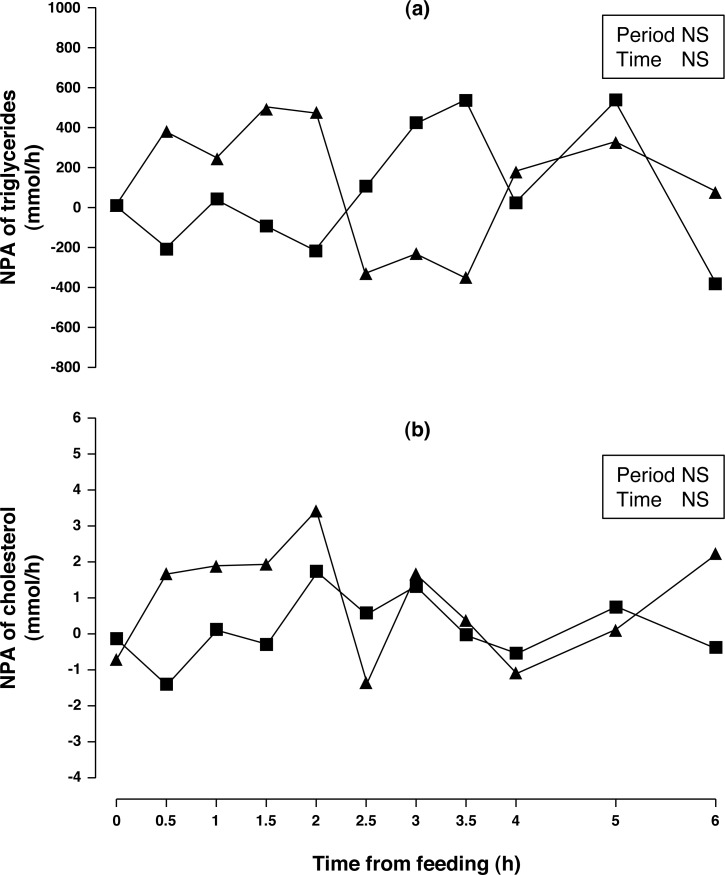
Net portal appearance (NPA) of triglycerides (A) and cholesterol (B) along a 6 h sampling in Iberian pigs (*n* = 6) fed acorns for 1 and 8 days (period 1 (■) and 2 (▴), respectively). NS, not significant (*P* > 0.10).

Portal and arterial concentrations and average NPA of metabolites are shown in Table 2. The progression along the sampling time of NPA is shown for ammonia, urea, glucose, lactate, albumin, creatinine, triglycerides and cholesterol (Figs. 1–4). PPF was greater in sampling period 2 compared to 1 (39%, *P* < 0.001; 841 and 607 mL/min, respectively). In addition, PPF was influenced by time (*P* < 0.05). The postprandial PPF peaks for sampling periods 1 and 2 were 0.5 h (with a maximum value of 860 mL/min) and 1 h (with a maximum value of 939 mL/min), respectively, and decreased thereafter to preprandial rate.

**Table 2:** Mean arterial and portal plasma concentrations, and net portal appearance (NPA) of metabolites in Iberian pigs (*n* = 6) after 1 and 8 d (periods 1 and 2, respectively) of acorns feeding.^a^

	Sampling period			*P*-value^b^		
	Period 1	Period 2	SEM^c^	Period	Time	Period × Time
Arterial (mmol/L)						
Ammonia	0.24	0.36	0.051	^***^	NS	NS
Urea	2.39	0.50	0.421	^***^	NS	NS
Glucose	4.64	4.89	0.221	^**^	NS	NS
Lactate	1.09	1.05	0.159	NS	^***^	NS
Albumin	0.56	0.52	0.025	^**^	NS	NS
Creatinine	0.054	0.062	0.003	^***^	NS	NS
Tryglycerides	0.38	0.38	0.083	NS	NS	NS
Cholesterol	2.13	2.33	0.145	^**^	NS	NS
Portal (mmol/L)						
Ammonia	0.42	0.41	0.051	NS	NS	NS
Urea	2.39	0.57	0.462	^***^	NS	NS
Glucose	5.83	5.91	0.303	NS	^***^	NS
Lactate	1.18	1.32	0.194	0.095	^**^	NS
Albumin	0.55	0.52	0.025	^**^	NS	NS
Creatinine	0.059	0.067	0.003	^***^	NS	NS
Tryglycerides	0.39	0.38	0.071	NS	NS	NS
Cholesterol	2.13	2.34	0.141	^**^	NS	NS
NPA (mmol/h)						
Ammonia	5.23	2.78	0.985	^***^	NS	NS
Urea	4.20	1.87	14.249	NS	NS	NS
Glucose	45.90	51.88	10.536	NS	^***^	NS
Lactate	4.36	14.43	3.217	^***^	^*^	NS
Albumin	−0.33	−0.28	0.327	NS	NS	NS
Creatinine	0.18	0.24	0.116	NS	NS	NS
Tryglycerides	0.73	1.18	0.815	NS	NS	0.088
Cholesterol	0.26	0.64	1.647	NS	NS	NS

**Notes.**

a Values are mean for ten postprandial measurements (0.5, 1, 1.5, 2, 2.5, 3, 3.5, 4, 5 and 6 h after feeding).

b Asterisks indicate significance at: * *P* < 0.05; ** *P* < 0.01; *** *P* < 0.001; NS, not significant (*P* > 0.10).

c Standard error of mean.

Arterial concentration of ammonia increased (50%, *P* < 0.01) in period 2 compared to period 1. No differences in portal concentration (*P* > 0.10) was found for ammonia between sampling periods. After one week of feeding the acorn diet, NPA of ammonia (Fig. 1A) decreased (47%, *P* < 0.05) over the 6 h sampling period. The portal ammonia balance accounted for 0.186 and 0.099 (0.143 on average) of total CP intake in periods 1 and 2, respectively. Arterial and portal concentrations of urea decreased (79 and 76%, respectively; *P* < 0.001) in sampling period 2 compared to sampling period 1 but no change in NPA of urea (Fig. 1B) was observed (*P* > 0.10). There was no time effect (*P* > 0.10) on concentrations and NPA of ammonia and urea.

Arterial concentration of glucose increased (5.4%, *P* < 0.01) although no differences in portal concentration and NPA (*P* > 0.10) were found between sampling periods. Portal concentration and NPA of glucose (*P* < 0.001) changed throughout time. Portal glucose peaked at 1.5 h and remained above basal level until 3 h after acorn consumption. NPA of glucose (Fig. 2A) peaked at 0.5 h and remained above basal level until 2.5 h after acorn consumption. Arterial lactate did not change (*P* > 0.10), while portal lactate tended to increase in sampling period 2 (12%, *P* = 0.095). Nevertheless, NPA of lactate (Fig. 2B) increased (231%, *P* < 0.001) after one week of feeding exclusively with acorns. Portal and arterial concentrations, and NPA of lactate peaked at 0.5 h and subsequently decreased to basal levels. Postprandial profiles of lactate and glucose were similar (the correlation between portal glucose and lactate concentration for periods 1 and 2 were *r* = 0.666 (*P* < 0.001) and *r* = 0.606 (*P* < 0.001), respectively); postprandial profiles of NPA of lactate and glucose were also similar (the correlation between NPA of glucose and lactate for periods 1 and 2 were *r* = 0.902 (*P* < 0.001) and *r* = 0.808 (*P* < 0.001), respectively).

Concentrations of arterial and portal albumin decreased (6.3%, *P* < 0.01) after consumption of acorns for one week (Table 2) while NPA of albumin (Fig. 3A) was negative, no change between periods was observed (*P* > 0.10). Arterial and portal concentration of creatinine increased (15 and 14%, respectively; *P* < 0.001) after one week of acorn consumption with no observed difference in NPA (Fig. 3B, *P* < 0.10).

Arterial and portal concentrations and NPA of triglycerides (Fig. 4A) were similar for both sampling periods (*P* > 0.10). The Period × Time interaction showed a trend (*P* = 0.088) for NPA of triglycerides. When NPA of triglycerides was evaluated through 2.5 h of sampling time, there was a significant increase (*P* < 0.05) in NPA after one week of acorn consumption. Arterial and portal concentration of cholesterol increased (9 and 10%, respectively; *P* < 0.01) after one week of acorn consumption but no change in NPA (Fig. 4B) was observed (*P* < 0.10).

## Discussion

In the present study growing Iberian pigs of 25 kg initial BW were used and first sampling took place at 34 kg BW. Although the use of a young pig as an appropriate model for a mature Iberian pig may be questioned, digestibility and balance studies performed in our Department (Nieto et al., 2002; García-Valverde et al., 2007) support the use of a growing Iberian pig as a model for heavier finishing pigs. Indeed, total tract apparent coefficients of digestibility for DM, organic matter and gross energy were similar in 50 and 104 kg BW Iberian pigs offered whole holm-oak acorns. Although the fermentative activity in the heavy pig must not be overlooked, at the low feeding level imposed in the present study similar NPA of nutrients can be expected irrespective of BW. Nevertheless, a slightly higher potential for protein accretion and lower lipid body deposition in the growing Iberian pig is likely to occur. The content of acorn tannins in the hull is approximately four times greater than in the kernel (Nieto et al., 2002). The pigs only consumed the inner kernels with an average tannin content of 10 g/kg DM, consuming much less tannins than the total acorn content. There is supporting evidence (García-Valverde et al., 2007) that in this context they are unlikely to influence digestion. In order to reproduce practical feeding management, pigs were trained to consume a quarter of their daily ration and the rest of it was offered after 6 h of sampling. In two previous studies, we decided to feed Iberian pigs an amount of feed proportional to the measurement period to better simulate the situation of Iberian pigs spending the whole day grazing to cover their nutritional requirements (Rodríguez-Estévez et al., 2010; González-Valero et al., 2016b). Similarly, Van der Meulen et al. (1997) measured NPA of nutrients in growing pigs offered the daily ration in two equal portions and sampled for 12 h after the first portion. We are aware that herbage, when available, represents the other feed resource in grazing pigs. However, the study focused on acorn consumption as it represents most of daily intake in grazing pigs (approximately 0.84 of ME (García-Valverde et al., 2007), or 0.9 of total dry matter (Rodríguez-Estévez et al., 2009)). This allowed for the shortening of experimental sampling time to avoid the intrinsic difficulties working with multi-catheterized pigs. With this background, the main objective of the present study was to evaluate the adaptation to an unbalanced low protein diet in Iberian pigs by examining the NPA of metabolites.

For the same level of intake, PBF for sampling period 1 was similar or slightly lower than Iberian pigs offered 145 or 160 g CP/kg diets, respectively (Rodríguez-López et al., 2010; González-Valero et al., 2016b). However, for sampling period 2, PBF was similar or slightly greater than Landrace pigs fed either 160 or 145 g CP/kg diet, respectively (Rodríguez-López et al., 2010; González-Valero et al., 2016b), at the same level of intake. When comparing PBF data in the literature, differences observed could be due to the experimental conditions or to the amount of feed intake. Hyperemia in the present study was probably diminished due to the low amount of feed offered before sampling (Fara, 1984), and to the low dietary protein content compared to previous studies with Iberian pigs (Rodríguez-López et al., 2010; González-Valero et al., 2016b). The reduced size of the meal (<15 g/kg BW) of very low CP content (52 g/kg DM) offered could partially explain the lower PPF since a relationship between feed intake and blood flow has been reported (Lomax & Baird, 1983; Huntington, 1984). In Iberian pigs we obtained differences in postprandial PBF related to the amount of feed intake; it was much lower when pigs were fed 0.25 (González-Valero et al., 2016b) than when they were offered the full daily ration (Rojas-Cano et al., 2017).

PPF was greater (*P* < 0.05) in sampling period 2 compared to 1 which may indicate a change in PDV physiology induced by an adaption to an acorn diet. Analogously, Yen & Nienaber (1992) observed a reduction in PBF and oxidative demand by the PDV in pigs seven days after switching from a standard to an antimicrobial-supplemented diet.

### Ammonia and urea

Portal was greater than arterial ammonia concentration as ammonia is converted into urea by the liver preventing hyperammonemia which would disrupt the central nervous system (Huizenga, Gips & Tangerman, 1996). There was evidence that NPA of ammonia was reduced after one week of adaptation to acorns in spite of similar portal concentration of ammonia. The NPA of ammonia results from enterocyte amino acid catabolism, namely aspartate, glutamine and glutamate (Windmueller & Spaeth, 1975; Windmueller & Spaeth, 1980; Remesy & Demigne, 1989) and microbial hydrolysis of circulating urea taken up by the intestine, as large intestine microflora is able to produce ammonia from dietary or endogenous nitrogenous compounds (Huizenga, Gips & Tangerman, 1996). Nevertheless, we were unable to detect a significant difference in NPA of urea (see below) between sampling periods, which might indicate that bacterial breakdown of urea into ammonia was similar or that ammonia from urea hydrolysis was used for *de novo* synthesis of bacterial protein (Mosenthin et al., 1992). In the present study, a decrease in NPA of ammonia must be explained by a decreased amino acid catabolism in first pass by the intestinal mucosa. Indeed, details of a decreased NPA of amino acids will be presented in the subsequent publication. When expressed relative to digestible protein intake, NPA of ammonia after one week of acorn consumption was half that of sampling period 1 (0.27 vs. 0.14). Reduced NPA of ammonia would save metabolic energy, as less ammonia would be available for conversion to urea, a process that requires ATP. We found no effect of sampling time on ammonia concentration or NPA, contrary to other authors that offered a much greater CP (160 g/kg) amount to the pigs (Yen & Pond, 1990).

The adaptation for one week to acorn feeding resulted in a decrease of plasma urea concentration of 4 mmol/L although no effect was found on the pattern of postprandial changes in plasma urea concentration. Lower plasma urea concentrations have also been reported in pigs when high compared to low fibre diets were fed, reflecting a larger transfer of fermentable carbohydrates to the large intestine (Malmlof, 1987) or when fermentation processes were increased after starch infusion at the distal ileum (Mosenthin et al., 1992). In our study, the diet was identical for both sampling periods and NPA of volatile fatty acids, the main end products of bacterial metabolism, was similar (González-Valero et al., 2016a) indicating comparable fermentation patterns in the hindgut. Luminal urea may be reabsorbed to the portal circulation or hydrolyzed to ammonia by bacterial ureases (Huizenga, Gips & Tangerman, 1996) that is either reabsorbed or used for bacterial protein synthesis. Decreased portal urea may be due to increased urea degradation to ammonia and subsequent use for bacterial protein synthesis; the reduced luminal concentration of urea after hydrolysis would explain a decreased reabsorption and subsequent appearance at the portal vein.

Coma, Carrion & Zimmerman (1995) reported that N retention and urea N concentration were reflective of one another such that urea N is minimized as N retention is maximized. Certainly, increased N retention, e.g., protein deposition, would depress the plasma urea concentration and urea excretion. Although we found lower plasma urea after one week of acorn feeding we did not measure N retention in our study. Amino acids and ammonia stimulate urea production in the liver (González-Valero et al., 2014), so decreased systemic urea would agree with the lower NPA of ammonia after one week of acorn consumption.

NPA of urea was positive in our study indicating a transfer of urea from the gastrointestinal tract to the circulation. In contrast, Iberian pigs fed a barley-soybean diet of greater CP (101 g/kg; Rojas-Cano et al., 2016) showed negative NPA of urea although diet composition in both studies differed in terms of both amino acid and fibre amount and profile. Indeed, it has been established that dietary type of fermentable energy and protein content affect urea concentration and NPA (Rérat & Buraczewska, 1986; Younes et al., 1995a; Younes et al., 1995b).

### Glucose and lactate

Portal glucose peaked at 1.5 h compared to maize or pea starch diets in white pigs (maximum glucose portal concentration at 0.5 and 1 h, respectively; Van der Meulen et al., 1997) and barley-soybean diets in Iberian pigs (maximum glucose portal concentration at 0.5 h; Rojas-Cano et al., 2016) indicating a slower digestion of acorn carbohydrates. A portion of acorn starch is resistant to ileal digestion (Morales et al., 2002) and enters the hind gut where fermentative degradation takes place, as we observed previously in Iberian pigs fed whole acorns (García-Valverde et al., 2007). However, calculations on previous work on glucose absorption kinetics in growing pigs showed greater rates of decrease in absorption after the maximum peak (8–10%/h (Rérat, Giusi-Perier & Vaissade, 1993; Van der Meulen et al., 1997)) to the one found in Iberian pigs fed acorns (5.3%/h on average 4.5 h after the glucose peak) in the present study. NPA of glucose did not change in period 2 in spite of decreased NPA of lactate. Whether lactate originated after glucose fermentation in the stomach and small intestine (Argenzio & Southworth, 1975; Cranwell, Noakes & Hill, 1976) or derived from glucose metabolism in the gut wall (Giusiperier, Fiszlewicz & Rérat, 1989) is not known. Although we cannot differentiate between microbial and metabolic lactate, most (0.87–0.93) of the lactic acid absorbed during the first 5 h after the intake of a diet containing different amounts of soluble and insoluble carbohydrates was L-lactic acid (Rérat, 1996). The very early appearance of lactate in the portal blood in our study seems to be in favor of increased glucose oxidation in the gut wall. Furthermore, the correlation between intestinal absorption of glucose and lactate may also indicate that lactate output is likely caused by a conversion of glucose to lactate by intestinal cells. Nevertheless, NPA of glucose was not decreased in sampling period 2, as it would be expected after increased glucose oxidation. It is possible that decreased glucose fermentation by intestinal microflora could have compensated lactate formation in the gut wall. Lactate is a product of postprandial glucose glycolysis (Mithieux, Rajas & Gautier Stein, 2004). Increased NPA of lactate after one week of acorn consumption will provide more precursors for hepatic gluconeogenesis. Indeed gluconeogenesis may be increased after one week of acorn consumption which is in line with the increased systemic glucose in period 2. As gluconeogenesis is an energy consuming process, this would imply augmented postprandial energy expenditure in Iberian pigs fed acorns for one week.

### Albumin and creatinine

Albumin concentration and NPA in Iberian pigs fed a barley-soybean meal diet of adequate CP (Rojas-Cano et al., 2016) were similar to what has been observed in the present study. Changes in serum albumin levels are relatively unspecific although hypoalbuminemias are observed in animals with malnutrition or deficient protein absorption (Kaneko, Harvey & Bruss, 2008). Although plasma proteins are sensitive to nutritional influences, the changes are often subtle and difficult to detect and interpret. It may be speculated that it takes at least one week of consumption of a very low protein (acorn) diet to develop hypoalbuminemia in the conditions of our study. Albumin is catabolized by all metabolically active tissues providing amino acids for the natural turnover of protein in peripheral tissues (Evans, 2002) and in line with this, negative NPA of albumin in Iberian pigs fed acorns indicates albumin utilization by the visceral tissue, may be as a source of amino acids or as an antioxidant (Anraku et al., 2001) although adaptation to acorn feeding did not alter the NPA. Albumin also functions as a circulating depot and transports molecules for a large number of metabolites including fatty acids (Evans, 2002).

Creatine is released from muscle in amounts proportional to muscle mass (Rassin & Bhatia, 1992) and it is the main source of the creatinine into the portal blood system (Kim et al., 2008). In our study, higher portal and arterial creatinine in pigs consuming acorns for one week was observed, but we do not think that it is a reflection of a greater muscle mass considering that dietary protein was below requirements. Instead, it is possible that substantial amounts of arginine, methionine and glycine were utilized in the liver to synthetize creatine and not used for protein synthesis, as a result of the lysine deficiency of acorn protein. Since dietary creatine is only provided in animal products, virtually all of the creatine loss in pigs must be replaced via endogenous synthesis, which may represent a considerable metabolic burden. Indeed, the methyl group for creatine synthesis is transferred from S-adenosylmethionine (Brosnan, Da Silva & Brosnan, 2011) the universal methyl donor originated from methionine. Nevertheless, there was no change in creatinine metabolism at the PDV level. Similar NPA of creatinine to this study were found in Iberian pigs fed a 101 g CP/kg DM barley-soybean meal diet (Rojas-Cano et al., 2016).

### Triglycerides and cholesterol

Contrary to protein and carbohydrates, absorbed fats are transported through the lymphatic system bypassing the portal vein and the liver, following digestion in the gastrointestinal tract. Although the portal system is commonly associated with transport of short and medium chain fatty acids, long chain fatty acids are also capable of being transported by the portal vein bound to albumin, albeit at a lower level (Weidman et al., 1980; Carlier & Bernard, 2000). Iberian pigs fed acorns (65.0 g fat/kg DM; present study) had greater plasma triglyceride concentration than Iberian pigs fed a sunflower oil diet of lower fat content (20 g/kg; Rojas-Cano et al., 2016), but with similar NPA of triglycerides. Diet composition has been reported to modulate plasma triglyceride content, so that it is increased by feeding high-fat diets (Mersmann et al., 1976) while the influence of dietary CP is controversial (Baker, Diller & Jordan, 1968; Madeira et al., 2016).

Although NPA of cholesterol was not altered, portal and arterial concentration slightly increased after one week of acorn consumption. Unfortunately, we have not measured any of the cholesterol fractions (HDL and LDL) that may have shown a different trend. Although it has been established that fat feeding (Allee, Baker & Leveille, 1971; Brooks et al., 1972; Mersmann et al., 1976) increases plasma cholesterol, Iberian pigs fed a 20 g sunflower oil/kg diet (Rojas-Cano et al., 2016) had similar cholesterol concentration and NPA to what was observed in the present study where fat intake was considerably greater.

## Conclusions

Adaptation to a low protein diet was evaluated in Iberian pigs. After one week of acorn consumption, pigs increased partial glucose oxidation at the PDV level while decreasing NPA of ammonia, which suggests diminished amino N catabolism in the enterocyte, thus saving metabolic energy as less ammonia would become available for conversion to urea.

##  Supplemental Information

10.7717/peerj.5861/supp-1 Data S1 Raw data used to calculate values displayed in the article, tables, and figures

## Additional Information and Declarations

### Data Availability

The following information was supplied regarding data availability: The raw data are provided in Data S1.

## References

[ref-1] Allee GL, Baker DH, Leveille GA (1971). Fat utilization and lipogenesis in the young pig. Journal of Nutrition.

[ref-2] Anraku M, Yamasaki K, Maruyama T, Kragh-Hansen U, Otagiri M (2001). Effect of oxidative stress on the structure and function of human serum albumin. Pharmaceutical Research.

[ref-3] Argenzio RA, Southworth M (1975). Sites of organic-acid production and absorption in gastrointestinal-tract of pig. American Journal of Physiology.

[ref-4] Association of Official Analytical Chemists (AOAC) (2000). Official methods of analysis.

[ref-5] Baker DH, Diller ER, Jordan CE (1968). Effect of a combination of diethylstilbestrol and methyltestosterone sex and dietary protein level on some serum lipids of finishing swine. Journal of Animal Science.

[ref-6] Brooks CC, Miyahara AY, Huck DW, Ishizaki SM (1972). Relationship of sugar-induced lesions in the heart of the pig to live weight, serum cholesterol and diet. Journal of Animal Science.

[ref-7] Brosnan JT, Da Silva RP, Brosnan ME (2011). The metabolic burden of creatine synthesis. Amino Acids.

[ref-8] Carlier H, Bernard A, Christophe AB, De Vriese S (2000). Chyloportal partition of fatty acids. Fat digestion and absorption.

[ref-9] Coma J, Carrion D, Zimmerman DR (1995). Use of plasma urea nitrogen as a rapid response criterion to determine the lysine requirement of pigs. Journal of Animal Science.

[ref-10] Cranwell PD, Noakes DE, Hill KJ (1976). Gastric secretion and fermentation in the suckling pig. British Journal of Nutrition.

[ref-11] Evans TW (2002). Review article: albumin as a drug—biological effects of albumin unrelated to oncotic pressure. Alimentary Pharmacology & Therapeutics.

[ref-12] Fara JW, Shepherd AP, Granger DN (1984). Postprandial mesenteric hyperemia. Physiology of the intestinal circulation.

[ref-13] García-Valverde R, Nieto R, Lachica M, Aguilera JF (2007). Effects of herbage ingestion on the digestion site and nitrogen balance in heavy Iberian pigs fed on an acorn-based diet. Livestock Science.

[ref-14] Giusiperier A, Fiszlewicz M, Rérat A (1989). Influence of diet composition on intestinal volatile fatty-acid and nutrient absorption in unanesthetized pigs. Journal of Animal Science.

[ref-15] Goering K, Van Soest PJ (1970). Forage fiber analyses (apparatus, reagents, procedures, and some applications). Agriculture handbook no. 379.

[ref-16] González-Valero L, Lachica M, Rodríguez-López JM, Lara L, Fernández-Fígares I, Skomial J, Lapierre H (2016a). Potential contribution of net portal absorption of volatile fatty acids to energy expenditure in Iberian gilts fed acorn.

[ref-17] González-Valero L, Rodríguez-López JM, Lachica M, Fernández-Fígares I (2014). Metabolic differences in hepatocytes of obese and lean pigs. Animal.

[ref-18] González-Valero L, Rodríguez-López JM, Lachica M, Fernández-Fígares I (2016b). Contribution of portal-drained viscera to heat production in Iberian gilts fed a low protein diet: comparison to landrace. Journal of the Science of Food and Agriculture.

[ref-19] Huizenga JR, Gips CH, Tangerman A (1996). The contribution of various organs to ammonia formation: a review of factors determining the arterial ammonia concentration. Annals of Clinical Biochemistry.

[ref-20] Huntington G (1984). Relationship of portal blood-flow to metabolizable energy-intake of cattle. Canadian Journal of Animal Science.

[ref-21] Kaneko JJ, Harvey JW, Bruss ML (2008). Clinical biochemistry of domestic animals.

[ref-22] Katz ML, Bergman EN (1969). Simultaneous measurements of hepatic and portal venous blood flow in the sheep and dog. American Journal of Physiology.

[ref-23] Kim JC, Mullan BP, Hampson DJ, Pluske JR (2008). Addition of oat hulls to an extruded rice-based diet for weaner pigs ameliorates the incidence of diarrhoea and reduces indices of protein fermentation in the gastrointestinal tract. British Journal of Nutrition.

[ref-24] Lomax MA, Baird GD (1983). Blood flow and nutrient exchange across the liver and gut of the dairy cow. British Journal of Nutrition.

[ref-25] López-Bote CJ (1998). Sustained utilization of the Iberian pig breed. Meat Science.

[ref-26] Madeira MS, Pires VMR, Alfaia CM, Lopes PA, Martins SV, Pinto RMA, Prates JAM (2016). Restriction of dietary protein does not promote hepatic lipogenesis in lean or fatty pigs. British Journal of Nutrition.

[ref-27] Malmlof K (1987). Porto-arterial plasma concentration differences of urea and ammonia nitrogen in growing pigs given high fiber and low fiber diets. British Journal of Nutrition.

[ref-28] Mersmann HJ, Allen CD, Steffen DG, Brown LG, Danielson DM (1976). Effect of age, weaning and diet on swine adipose-tissue and liver lipogenesis. Journal of Animal Science.

[ref-29] Mithieux G, Rajas F, Gautier Stein A (2004). A novel role for glucose 6-phosphatase in the small intestine in the control of glucose homeostasis. Journal of Biological Chemistry.

[ref-30] Morales J, Pérez JF, Baucells MD, Mourot J, Gasa J (2002). Comparative digestibility and lipogenic activity in Landrace and Iberian finishing pigs fed ad libitum corn- and corn-sorghum-acorn-based diets. Livestock Production Science.

[ref-31] Mosenthin R, Sauer WC, Henkel H, Ahrens F, De Lange CF (1992). Tracer studies of urea kinetics in growing pigs: II. The effect of starch infusion at the distal ileum on urea recycling and bacterial nitrogen-excretion. Journal of Animal Science.

[ref-32] Nieto R, Lara L, Barea R, García-Valverde R, Aguinaga MA, Conde-Aguilera JA, Aguilera JF (2012). Response analysis of the Iberian pig growing from birth to 150 kg body weight to changes in protein and energy supply. Journal of Animal Science.

[ref-33] Nieto R, Rivera M, García MA, Aguilera JF (2002). Amino acid availability and energy value of acorn in the Iberian pig. Livestock Production Science.

[ref-34] Rassin DK, Bhatia J, Nissen S (1992). Evaluation of protein status in humans. Modern methods in protein nutrition and metabolism.

[ref-35] Remesy C, Demigne C (1989). Specific effects of fermentable carbohydrates on blood urea flux and ammonia absorption in the rat cecum. Journal of Nutrition.

[ref-36] Rérat A (1996). Influence of the nature of carbohydrate intake on the absorption chronology of reducing sugars and volatile fatty acids in the pig. Reproduction, Nutrition, Development.

[ref-37] Rérat A, Buraczewska L (1986). Postprandial quantitative kinetics of urea and ammonia nitrogen exchanges between the digestive tract and the portal blood in conscious pigs receiving a diet with or without urea. Archiv fur Tierernahrung.

[ref-38] Rérat A, Giusi-Perier A, Vaissade P (1993). Absorption balances and kinetics of nutrients and bacterial metabolites in concious pigs after intake of maltose- or maltitol-rich diets. Journal of Animal Science.

[ref-39] Rivera-Ferré MG, Aguilera JF, Nieto R (2005). Muscle fractional protein synthesis is higher in Iberian than in landrace growing pigs fed adequate or lysine-deficient diets. Journal of Nutrition.

[ref-40] Rodríguez-Estévez V, García A, Peña F, Gómez AG (2009). Foraging of Iberian fattening pigs grazing natural pasture in the dehesa. Livestock Science.

[ref-41] Rodríguez-Estévez V, Sánchez-Rodríguez M, García A, Gómez-Castro AG (2010). Feed conversion rate and estimated energy balance of free grazing Iberian pigs. Livestock Science.

[ref-42] Rodríguez-López JM, Lachica M, González-Valero L, Fernández-Fígares I (2010). Energy expenditure of splanchnic tissues in Iberian and Landrace growing gilts. Livestock Science.

[ref-43] Rodríguez-López JM, Lachica M, González-Valero L, Fernández-Fígares I (2013). Approaches for quantifying gastrointestinal nutrient absorption and metabolism in a native and a modern pig breed. Journal of Agricultural Science.

[ref-44] Rojas-Cano M, Fernández-Fígares I, Lara L, Lachica M (2016). Influence of betaine and conjugated linoleic acid on portal-drained viscera flux of metabolites in growing Iberian pigs. Journal of Animal Science.

[ref-45] Rojas-Cano M, Lachica M, Lara L, Haro A, Fernández-Fígares I (2017). Portal-drained viscera heat production in Iberian pigs fed betaine- and conjugated linoleic acid-supplemented diets. Journal of the Science of Food and Agriculture.

[ref-46] Statistical Analysis System (SAS Institute) (2002). SAS user’s guide: statistics.

[ref-47] Van der Meulen J, Bakker JGM, Smits B, DeVisser H (1997). Effect of source of starch on net portal flux of glucose, lactate, volatile fatty acids and amino acids in the pig. British Journal of Nutrition.

[ref-48] Weidman M, Fisher L, McDonald GB, Saunders DR (1980). Portal venous transport of long-chain fatty acids absorbed from rat intestine. American Journal of Physiology.

[ref-49] Windmueller HG, Spaeth AE (1975). Intestinal metabolism of glutamine and glutamate from lumen as compared to glutamine from blood. Archives of Biochemistry and Biophysics.

[ref-50] Windmueller HG, Spaeth AE (1980). Respiratory fuels and nitrogen metabolism in vivo in small intestine of fed rats. Quantitative importance of glutamine, glutamate, and aspartate. Journal of Biological Chemistry.

[ref-51] Yen JT, Killefer J (1987). A method for chronically quantifying net absorption of nutrients and gut metabolites into hepatic portal vein in conscious swine. Journal of Animal Science.

[ref-52] Yen JT, Nienaber JA (1992). Influence of carbadox on fasting oxygen consumption by portal vein-drained organs and by the whole animal in growing pigs. Journal of Animal Science.

[ref-53] Yen JT, Pond WG (1990). Effect of carbadox on net absorption of ammonia and glucose into hepatic portal vein of growing pigs. Journal of Animal Science.

[ref-54] Younes H, Demigne C, Behr S, Remesy C (1995a). Resistant starch exerts a lowering effect on plasma urea-by enhancing urea N transfer into the large-intestine. Nutrition Research.

[ref-55] Younes H, Garleb K, Behr S, Remesy C, Demigne C, Rémésy C, Demigné C (1995b). Fermentable fibers or oligosaccharides reduce urinary nitrogen excretion by increasing urea disposal in the rat cecum. Journal of Nutrition.

[ref-56] Zierler KL (1961). Theory of use of arteriovenous concentration differences for measuring metabolism in steady and non-steady states. Journal of Clinical Investigation.

